# Substance Use, Mental Health and Healthcare Among Young Adults with Adverse Childhood Experiences During COVID-19

**DOI:** 10.1007/s40653-026-00923-1

**Published:** 2026-06-29

**Authors:** Sheila Pakdaman, Shaddy K. Saba, Jennifer Weck, Jane Steinberg, Jessica L. Barrington-Trimis, Jordan P. Davis, John D. Clapp, Jennifer B. Unger

**Affiliations:** 1 Department of Population and Public Health Sciences, University of Southern California, Los Angeles, CA 90089 USA; 2 USC Institute for Addiction Science, Los Angeles, CA 90032 USA; 3 Suzanne Dworak-Peck School of Social, University of Southern California, Los Angeles, CA 90089 USA

## Abstract

Young adults with Adverse Childhood Experiences (ACE), e.g., household dysfunction and maltreatment) are at high risk for poor health outcomes such as mental health and substance use disorders. The COVID-19 pandemic imposed numerous external stressors and impacted access to mental healthcare services for many individuals. This study explores mental health, substance use and mental healthcare experiences among ACE-exposed young adults during the COVID-19 pandemic. Young adults (*N* = 26, ages 21–29 years old, 15 males, 9 females, and 2 non-binary) who experienced ACE were recruited online to learn about their histories of substance use, anxiety, depression, and mental healthcare experiences during COVID-19. After interviews via Zoom, transcription was completed and thematic analysis was conducted. Thematic analyses revealed several factors impacting young adults’ substance use and mental health behaviors during the pandemic. Young adults with a history of ACE experienced the following: isolation, grief and loss, financial and employment disruption, problematic interpersonal relationships that were exacerbated by staying home, and health-related anxiety. Taken together, these experiences impacted their substance use, mental health and mental healthcare services. Young adults who experienced ACE indicated that substances helped alleviate feelings of anxiety and depression. However, young adults who received mental healthcare during the pandemic reported that such care benefited them in reducing symptomology. Future pandemic responses should include a focus on mental healthcare for young adults at risk for substance use and mental health problems.

**Supplementary Information:**

The online version contains supplementary material available at https://doi.org/10.1007/s40653-026-00923-1.

## Introduction

Adverse childhood experiences (ACE) are traumatic events that occur during childhood, including homelessness; abuses; parental intimate partner violence; household substance abuse; divorce, and familial incarceration (CDC, 2019; O’Malley, 2004; SAMSHA, 2019). Young adults who experienced ACE are considered a high-risk population because they are more likely to engage in substance misuse, (CDC, 2019; Masten & Wright, 1998; NIAAA, 2019). have higher rates of anxiety and depression, (Austin & Sutton, 2014; Bambra et al., 2020; Barnes et al., 2022) are more likely to need psychiatric care, (Coley & Baum, 2022; Collier Villaume et al., 2023) and have higher overall healthcare costs, (Emery et al., 2021) compared to their non-ACE peers. A recent systematic review concluded that the strong association between ACE and later mental health problems calls for better intervention strategies (McKay et al., 2021). The COVID-19 pandemic might have exacerbated mental health challenges among young adults who experienced ACE (Watson et al., 2020). Thus understanding the relationship between mental health, substance use, and mental healthcare for young adults who experienced ACE during the COVID-19 pandemic is instrumental for this population.

Early adulthood represents a critical developmental period marked by identity formation, increasing independence, and transitions in education, employment, and healthcare responsibility (Benson & Furstenberg, 2006; Watson, et al., 2020). During this life stage, many individuals age out of pediatric or family-based healthcare systems and must independently navigate mental healthcare access and utilization.(Gray et al., 2018; Khetarpal et al., 2022; Rosina et al., 2025). These transitions can be particularly challenging for young adults who have experienced ACE, as early trauma has been linked to difficulties with emotional regulation, coping, and help-seeking behaviors (Danese and McEwen 2012; Felitti et al. 1998a; McLaughlin et al. 2011). The convergence of developmental vulnerability and elevated risk for mental health and substance use problems makes early adulthood a particularly salient period for examining the long-term impacts of ACE, especially in the context of an acute global stressor such as the COVID-19 pandemic.

Stressors such as academic, familial, and societal problems increased worldwide during the COVID-19 pandemic. The World Health Organization reported that young adults experienced a disproportionate decline in mental health during the COVID-19 pandemic compared to other age groups, representing a marked increase in psychological distress relative to pre-pandemic levels (World Health Organization, 2022). Young adults faced heightened academic, economic, and social stressors, alongside widening gaps in mental health and substance use treatment access during this period (Graupensperger et al. 2021a, b; Russell et al. 2024; Wiedemann et al. 2024). Despite the rise in telemedicine during the COVID-19 pandemic, (Hincapié et al., 2020) young adults reported a lack of access to mental health services due to financial barriers (Hincapié et al., 2020) lack of health insurance, and poor treatment effectiveness (Reay et al., 2020). Many of these barriers, including financial constraints, lack of health insurance, and perceived poor treatment effectiveness, existed prior to the pandemic (2016), but were exacerbated during COVID-19 due to widespread service disruptions and increased demand for care (Coley & Baum, 2022; Stepanova et al., 2024).

Declines in mental health, in turn, may exacerbate underlying conditions, especially for young adults who have experienced ACE and are predisposed to mental health disorders and problematic substance use. In the United States, where mental healthcare access is largely dependent on insurance status, financial resources, and geographic availability of services, young adults may face unique barriers to care, particularly during periods of widespread disruption such as the COVID-19 pandemic (2022). Previous studies have examined substance use behaviors and mental health among young adults who experienced the COVID-19 pandemic (Alradhi et al. 2022; Graupensperger et al. 2021a, b; McManus and Ball 2020; Romm et al. 2021). However, few COVID-19 studies have focused on young adults who experienced ACE. Qualitative interviews are beneficial in understanding the experiences of individuals with a history of trauma (Barnes et al., 2022), as well as young adult substance use and mental health behaviors (Lyons et al., 2015; McMillan et al., 2022). Exploratory qualitative studies provide a means for understanding the “how” and “why” in research (Cleland, 2017). Using a combination of both deductive and inductive approaches (Bingham et al., 2022; Edmonds & Warburton, 2016) allows for the creation of a theoretical framework to guide the research question (deductive) while also enabling the coding process to begin after analyzing the interview transcripts (inductive). This combined approach is organized and thorough (Bingham et al., 2022), which can help us better understand how a new virus, such as COVID-19, can impact behavior. Adopting a qualitative approach can provide insight into how mental healthcare experiences influenced the substance use and behaviors of young adults who have experienced ACE and why these behaviors differed from the pre-pandemic period.

The *Transactional Model of Stress and Coping* (Lazarus & Folkman, 1987) posits that events are experienced as stressful based on how the individual perceives them, including the individual’s sense of the intensity of the stressor and their cognitive capacity for responding (Alradhi et al., 2022; Bellis et al., 2022; Lazarus & Folkman, 1987; Slone et al., 2022; World Health Organization, 2022). This theory suggests that individual’s responses to the pandemic will vary based on their own cognitive capacities (Folkman et al., 1986). Thus, young adults with ACE who perceive that they have inadequate resources to cope with the pandemic may experience relatively high levels of depression and anxiety (Na et al., 2015). To mitigate the feelings of depression and anxiety, such an individual may be at risk for poor coping behaviors such as substance use (Na et al., 2015). Conversely, those who believe that they have sufficient resources to cope with the pandemic may be more likely to exhibit resiliency (Zimmerman, 2013) to substance use and mental health problems.

## Current Study

Research on the mental healthcare experiences of young adults who experienced ACE during the current COVID-19 pandemic is limited. Given the developmental significance of early adulthood and the unique structure of the U.S. healthcare system, the current study investigates how young adults who experienced ACE have been impacted during the COVID-19 pandemic, focusing on their mental health, mental healthcare access and utilization, and substance use behaviors. Based on the *Transactional Model of Stress and Coping *(Lazarus & Folkman, 1987), young adults who experienced ACE had an elevated risk for anxiety, depression, substance use, and need for mental healthcare before the pandemic, (Felitti et al. 1998a) and these problems increased worldwide during the pandemic (Alradhi et al., 2022; Anderson et al., 2022). It is therefore critical to understand the pandemic’s effect on this vulnerable population as there may be residual aftereffects and the potential for mental healthcare services to ameliorate the pandemic’s negative effects.

Specifically, this study explores (1) how pandemic-related stressors influenced participants’ mental health and substance use behaviors, (2) how access to and experiences with mental healthcare shaped coping responses during the pandemic, and (3) how young adults perceived the role of mental healthcare in mitigating or exacerbating changes in mental health and substance use compared to the pre-pandemic period. By qualitatively examining these interconnected processes, the study aims to elucidate the mechanisms through which stress appraisal, coping resources, and healthcare access interact to influence behavioral and mental health outcomes among young adults with ACE, using both deductive and inductive strategies. The theoretical framework of the *Transactional Model of Stress and Coping* (Lazarus & Folkman, 1987) is used to guide the research aim deductively, while the inductive strategy involves coding after reading over the interview transcripts (Bingham et al., 2022; Edmonds & Warburton, 2016). Using a geographically diverse sample of young adults across the United States, this study seeks to capture a range of experiences shaped by differing regional and healthcare contexts. Moreover, the focus of this study is to explore the experiences of young adults who have a history of ACE during the COVID-19 pandemic, which was a global traumatic experience. This study uniquely integrates participants lived experiences of trauma, stress appraisal, mental healthcare access, and coping behaviors to examine how these factors collectively shaped mental health and substance use outcomes during a global crisis. The ultimate objective of this research is to gain insights that can inform future mental health and healthcare approaches as well as substance use interventions for young adults with a history of ACE, beyond the context of the COVID-19 pandemic.

## Methods

### Participants & Procedures

Using qualitative interviewing, 26 young adults who experienced ACE were interviewed to learn about their substance use, anxiety, depression, and their subjective mental healthcare experiences during the COVID-19 pandemic. Participants residing in the United States were recruited using online advertisements and community-based flyers. Online recruitment was conducted via LinkedIn, X (formerly Twitter), and Instagram using posts targeted toward users interested in health, mental health, and substance use-related content. In addition, flyers were distributed at outpatient social service and behavioral health organizations in the United States, including community mental health centers and counseling/substance use treatment agencies serving individuals with current or prior mental health and substance use concerns. Recruitment materials described the study as a research interview on mental health, substance use, and coping behaviors among young adults who experienced childhood trauma. Because recruitment occurred through social media and service-providing organizations, the sample is likely to overrepresent individuals already engaged with or interested in mental health and substance use topics. Interested participants completed a screener to ensure they qualified for the study. The inclusion criteria included being within the age range of 21–29, as we studied substance use behaviors and needed participants to be of legal age. Also, participants had to have experienced at least one ACE. Afterwards, eligible participants were sent an informed consent form to sign online and were invited to participate in a 30-to-45-minute one-on-one interview via Zoom. From January through April 2022, participants were interviewed to understand their subjective experiences before and during COVID-19. The number of interviews conducted was selected based on previous studies until data saturation was achieved (Francis et al., 2010; Guest et al., 2006, 2020, p. 20). All interviews took place via Zoom and were conducted by the lead author and a co-author with experience conducting qualitative interviews. Participants were given a gift card for their time and participation in the study.

## Measures

The interview questions are shown in Supplementary Table 1. Interviews were conducted by the lead author, a young adult Middle Eastern cisgender woman who completed this study as part of her doctoral dissertation and received formal training in qualitative research methods and trauma-informed group facilitation during her PhD program, and by a Master of Social Work research assistant, an adult White cisgender woman with training in qualitative methods and trauma-informed approaches through both clinical practice and relevant coursework supporting this study. The overall study was conducted under the supervision of the doctoral mentor, a middle-aged White cisgender woman with ACE exposure who remained attentive to how social distance and positional power might shape interpretation and therefore emphasized grounding analytic claims in participants’ own words and meanings. The research team acknowledges that researchers’ identities, training, and experiences may influence data collection, analysis, and interpretation. Participants first responded to demographic questions and the ACE questionnaire verbally to confirm their ACE exposure and build rapport with the interviewer. Next, they responded to semi-structured interview questions about the effects of their ACE, (Felitti et al. 1998b) substance use, mental health, and mental health experiences before and during the COVID-19 pandemic. These interviews were focused on understanding (1) any feelings of anxiety or depression that may or may not be about the pandemic (2) participants’ use of and experiences with mental healthcare and (3) problematic substance use behaviors during the pandemic and how participants’ substance use related to their mental health.

### Thematic Analyses

Interviews were analyzed using thematic analysis (Boyatzis, 1998; Roulston, 2001), which is widely used in health sciences studies (Braun & Clarke, 2006; Guest et al., 2012). Thematic analyses allows for suitability and flexibility for identifying patterns across participants’ lived experiences. This approach was appropriate for examining how young adults with a history of ACE understood and navigated mental health, substance use, and mental healthcare access during the COVID-19 pandemic. Data compiling (Castleberry & Nolen, 2018) was completed, where the interviews were transcribed using a professional transcription service. Next, disassembling (Castleberry & Nolen, 2018) of the transcriptions took place, in which they are read line by line to uncover themes, concepts, or ideas that may be connected (Austin & Sutton, 2014). Interview transcripts were read over, corrected for accuracy, and uploaded to Atlas.ti version 22 (ATLAS.Ti Scientific Software Development GmbH [ATLAS.Ti 22 Mac], 2022). Next, data were reassembled (Castleberry & Nolen, 2018) where overarching themes were created based on participant interview data, (Braun & Clarke, 2006) using inductive coding (Bingham et al., 2022). This allowed for the integration of deductive coding guided by *The Transactional Model of Stress and Coping* (Lazarus & Folkman, 1987) alongside inductive coding grounded in the data. This process continued until themes were saturated and no new themes were identified (Austin & Sutton, 2014). A codebook was created allowing all data analysts to work from the same framework during the coding process. The codebook was organized by identified themes and their corresponding codes, such as childhood trauma, substance use, mental health, mental healthcare and fears associated with COVID-19. Coding was conducted by the lead author and three co-authors. An intra-coder kappa reliability score of at least 0.85 was used to determine that analysts were in agreement discussed and came to a consensus if disagreements arose (Golafshani, 2003). Next, interpretation (Castleberry & Nolen, 2018) was completed, in which the major themes were identified to understand and discuss the findings. This included considering the relationship between themes and any global findings between the coders. Lastly, concluding (Castleberry & Nolen, 2018) was completed, in which the research questions are answered and conclusions and implications are generated (Yin, 2016). To minimize bias, reflexive memoing, coding team-based discussions, and iterative codebook refinement were used throughout the analytic process. Table 1 shows the themes and their definitions.

**Table 1:** Themes and definitions

Themes	Definitions
Mental health & factors that impacted mental health during COVID-19	Participants described their mental well-being during the COVID-19 pandemic compared with pre-pandemic.
Substance use patterns & factors that impacted substance use during COVID-19	Participants described their substance use behaviors during the COVID-19 pandemic and how it differed from prior to the pandemic.
Healthcare & coping strategies for mental health and substance use during COVID-19	Participants described their mental healthcare experiences and specific coping mechanisms used for mental health and substance use (e.g., sleep, exercise, gaming).

## Results

### Participant Demographics and ACE

Participants’ mean age was 23.51 (SD = 2.41); 15 were male, 9 female, and 2 non-binary. Participants reported a mean of 4.5 ACE, including a median of 2 types of maltreatment and 3 types of household dysfunction prior to the age of 18. The majority of participants were either enrolled in school and/or employed and were also geographically distributed across the United States, with 15 residing on the West Coast, 8 on the East Coast, and 3 in the Midwest.

### Themes

#### Mental Health & Factors That Impacted Mental Health During COVID-19

All participants reported experiencing mental health symptomology during the COVID-19 pandemic. Describing their depression, a participant stated, “I ended up sleeping a lot and I really didn’t have any motivation to do anything. There wasn’t really a point, so I guess that would be hopelessness” (Participant #13). Similarly, many participants experienced anxiety frequently during the pandemic, with many experiencing anxieties daily or almost every day. “I developed kind of a hair-pulling thing,” explained Participant (#2). In fact, nearly all young adult participants reported an increase in mental health problems during COVID-19.

Participants often described being affected by social isolation and loneliness during the pandemic. Also, participants often described the challenge of having to cancel holiday plans, not seeing friends or family, and being stuck indoors for long periods of time. One participant explained how they felt that they no longer had a social life. Another described how their ACE history may have placed them at particular risk of being affected by social isolation and loneliness during COVID-19: “Being lonely and just dreading in my thoughts about my childhood and all that literally came back to me when I was alone during COVID” (Participant #14). In fact, participants often noted that during re-openings and periods when they *were* able to see loved ones, their mental health greatly improved. On the other hand, some participants reported that socializing less and having less to do during the pandemic helped them feel *less* anxious, with one noting that socializing via technology seemed to benefit their mental health.

Young adults reported other COVID-19-related interpersonal issues, including romantic break-ups during the pandemic, challenges they faced living with family members, and difficulty navigating differences in preferences around social distancing measures: “A couple months in, when people were starting to get together more, I felt more anxious because I felt pressured to spend time with people. And I wanted to. But at the same time didn’t think that it was a good idea. And I did have a lot of anxiety saying no to family members and saying no to my friends, worrying about how that was gonna affect our relationships” (Participant #26).

Most participants experienced some health-related anxiety during the pandemic, namely fear of themselves or others catching COVID-19. Health anxiety contributed to the reduction in social activity for many interviewees, including a participant who described the link in the following way: “I think the disease affected little things, how we interact with people, and also, we don’t know if someone is exposed to COVID-19, so it was really hard. It was really hard” (Participant #3). Some participants were especially worried about their or others’ pre-existing medical conditions and feared this would put them at risk of poor outcomes if they contracted the virus.

Participants often specifically noted a fear of themselves or others dying due to the pandemic, and several participants did in fact experience a death or other loss during the pandemic and identified a link between grief and their mental health difficulties. Although the emergence of vaccines helped assuage fears, many participants had fears about the vaccines that created more anxiety. Worries about the vaccine included having more severe symptoms of COVID-19 *after* receiving the vaccine, the vaccine affecting childbearing abilities, or even dying due to the vaccine.

Participants often also had financial or employment-related concerns that affected their mental health. Participants discussed the effect of frequent job changes, losing work, and pay cuts that they or their loved ones experienced. A participant who suffered a job loss stated, “I was depressed because I lost my job. My main source of income during the pandemic” (Participant #5).

#### Substance Use Patterns & Factors That Impacted Substance Use During COVID-19

Alcohol was the most frequently used substance and was used by nearly all young adult participants specifically during the pandemic. Many participants reported drinking nearly every day and/or binge drinking. A participant illustrated the extent of their heavy drinking by saying, “I was just drinking a lot. I was always drinking. I was putting a little bit of stuff in my coffee in the morning, but I was not becoming drunk, I just needed it to get through the day. But then at night I was drinking quite a bit and that probably lasted for like a month, I guess” (Participant #6). In terms of other substances, over half of the participants reported some cannabis use, and many of these participants reported daily or nearly daily use of cannabis during the pandemic. A minority of participants endorsed other illicit substance use or prescription drug misuse during the pandemic. Considering all substances, nearly all described increases in use behaviors during COVID-19. In fact, some participants expressed concern that their substance use was becoming addictive. One, for example, stated, “I would say, during the pandemic, I got addicted to taking alcohol…I have to smoke marijuana early in the morning once I get out of bed” (Participant #7).

The factors that influenced participants’ mental health also influenced their substance use behaviors. However, because mental health and substance use represent distinct yet related constructs, they are discussed separately to highlight their unique characteristics and implications. Nearly all participants reported that their use of substances during COVID-19 was in part an attempt to cope with mental health symptomology, with several participants specifically noting they used substances to cope with depression and anxiety. Describing how alcohol seemed to help with their anxiety symptomology, one participant stated, “It’s just, I think, a nice way to calm down and think about something else, because it kind of slows down thoughts, so that helps” (Participant #23). Several participants said they used substances to slow their mind down or relax enough to fall asleep when their mental health symptomology made it difficult to do so. However, one participant shared that their attempts to self-manage their mental health symptomology with substances were not effective: “I wanted to add that my use of substances didn’t, in any way, give me a permanent relief. For that moment, two hours, sometimes 30 minutes, I could feel relief, I could feel free. After which, those same things I tried to run away from, come again” (Participant #5). Another seemed to feel that the use of substances was making them feel worse, stating substances impacted their mental health negatively. Describing the potentially reciprocal links between substance use and mental health, another participant stated “I’m not sure what happens first; like if the depression is what triggers that or if the substance use is what triggers the lengthy depression crashes. But they definitely coincide for sure” (Participant #18). Additionally, some participants linked their substance use to experiences of death and loss during the pandemic. One shared, “Obviously being exposed to so much death, it just – I would go reach for the drink to erase this; this pain out of my head and out of my memory” (Participant #6).

#### Healthcare & Coping Strategies for Mental Health and Substance Use During COVID-19

Although all participants experienced some degree of mental health symptomology and many reported problematic substance use during the pandemic, only some participated in some form of mental healthcare (i.e., therapy or psychiatric care). Of those who received mental healthcare services, most felt that doing so benefited them in reducing or preventing their mental health symptomology, as they often stated that they likely would have fared worse during the pandemic had they not engaged in treatment. Participants often utilized telehealth during the pandemic, and many reported that they found telehealth particularly helpful and convenient. Describing how telehealth lowered barriers, one participant shared, “Having [sessions] be virtual makes it really easy for me to incorporate it into my day, even if I really don’t wanna do it that day” (Participant #26).

##### Service Challenges

However, other participants found telehealth challenging, noting glitching (malfunctioning telehealth software), privacy concerns, and awkwardness. One participant described feeling self-conscious on group sessions via Zoom, sharing that it made her nervous. Another participant felt individual sessions with her psychologist via telehealth were inferior to those she has had in person “because he understands my body language [in person]. So, I feel that meeting him in-person is better” (Participant #7). Notably, nearly some participants reported they did not have health insurance or could not afford care during the pandemic. Describing their predicament, one participant stated, “I would like to but it’s just the fact that the services are pretty expensive for me right now and I just can’t justify the expense” (Participant #15). One participant who did not have adequate insurance coverage stated that, instead, her friend group had formed a self-help group to cope with the challenges of the pandemic.

##### Coping Strategies

Participants identified a number of other coping strategies they had been utilizing during the pandemic. Most frequently, participants spoke of exercise as a way to feel better, and one of these participants shared that they used exercise as a “healthy distraction from drinking” (Participant #11). Several participants described their use of mindfulness or self-compassion skills that they had learned either through therapy or with a mindfulness app, and some spoke highly of the benefits.

## Discussion

This study examined how young adults who experienced ACE fared during the COVID-19 pandemic by interviewing them about their substance use, mental health, and mental healthcare during this time. As the COVID-19 pandemic was an unprecedented, shared experience, it provides a unique opportunity to study young adults who experienced ACE, who are already at high risk for the aforementioned problems, during a global traumatic event. Several common themes were identified in this analysis, including factors that impacted mental health and substance use behaviors of young adults who experienced ACE during the pandemic, as well as mental healthcare and other means of coping during the COVID-19 pandemic.

Overall, most young adults in this sample indicated that they experienced depression and/or anxiety during the COVID-19 pandemic (Collier Villaume et al., 2023; Varma et al., 2021; Xiang et al., 2024). This aligns with what has been found in the recent COVID-19-related literature, that young adults who experienced ACE are more susceptible to mental health complications during traumatic conditions than compared to young adults without ACE (Castellini et al., 2022; Li et al., 2021; Romm et al., 2021; Rosenthal et al., 2021). However, our study found that several factors impacted mental health for our participants, including loneliness and isolation (Boyraz et al., 2020; Zhao et al., 2020), and some reported their difficulty was further exacerbated by the effect of previous trauma (Guadagni et al., 2020; Slone et al., 2022). This indicates that previous trauma can have an impact on future trauma responses. Young adults indicated that seeing loved ones in-person or talking to them via technology (Cao et al., 2023; Pakdaman & Clapp, 2021) helped alleviate feelings of loneliness and isolation during the pandemic, which is indicative of counteracting loneliness with connectedness, even via technology. Future studies should focus on understanding the relationship of technology and connectivity, especially in young adults with trauma, and potential mental health implications. In line with *The Transactional Model of Stress and Coping* (Lazarus & Folkman, 1987) we found that having coping strategies does impact the stress response of young adults with a history of ACE during the COVID-19 pandemic and tapping into those coping strategies when appropriate can help with stress response accordingly.

Additionally, other participants stated that their peer groups and loved ones were not understanding if they expressed anxiety about the COVID-19 pandemic and in-person interactions. In line with this, Chou and colleagues found that social distancing was not widely supported, meaning that peers did not follow the government-recommended social distancing measures (Chou et al., 2020). Young adults tend to socialize in groups; thus, it is probable that those social distancing measures were not followed as strictly as needed. An interesting finding of our study is that many participants reported other interpersonal issues that arose during the pandemic and due to social distancing measures. For example, the COVID-19 pandemic required many to spend a lot of time with family members they normally do not cohabitate with and in some cases with whom they experienced relational strain or difficulty (e.g., college students sometimes had to move back to their family home during campus lockdowns, and back with their family of origin where they experience childhood trauma). It is possible that some of this conflict was because social distancing measures made it difficult for young adults to socialize outside of their homes (Lee et al., 2024; Sinko et al., 2022), and have independence to live without restrictions.

Health anxiety was also a common theme in our study, though it was experienced in differing ways. On one hand, many of our young adult participants were concerned about catching the COVID-19 virus or having a loved one contract the virus. Many even reported a fear of dying from the COVID-19 virus (Son et al., 2020), which other studies have found to be the case for young adults who did not experience ACE (Pradhan et al., 2022). Young adults with a history of ACE and elevated anxiety may also be more likely to experience heightened health anxiety. In line with *The Transactional Model of Stress and Coping*, (Lazarus & Folkman, 1987) the way an individual perceives and evaluates a situation, such as experiencing health anxiety during a pandemic, can determine their stress response. When it came to the COVID-19 vaccine, some young adults were relieved and saw it as a solution to their anxiety (Graupensperger et al. 2021a, b), but others worried the vaccine would cause severe adverse reactions such as problems with fertility and even death (Gurley et al., 2021; Saied et al., 2021). Bellis and Colleagues reported that young adults (aged 18–29) had the highest percentage of vaccine hesitancy when compared to other adult age groups (Bellis et al., 2022). Fears associated with the vaccine was an interesting finding of our study, which is in line with other literature on the topic of ACE and vaccine hesitancy behaviors (Afifi et al., 2021). Future public health and pandemic responses should focus on interventions to reduce vaccine hesitancy in vulnerable populations, such as young adults who have experienced ACE, who may potentially be more susceptible to health anxiety.

Another factor that impacted mental well-being during the COVID-19 pandemic was financial stressors (Knell et al., 2020). Moreover, finances have greatly impacted many individuals during the COVID-19 pandemic, in particular young adults (Chase et al., 2024; Hincapié et al., 2020; Knell et al., 2020). This study adds to the literature, by taking a qualitative approach to understanding the financial stressors that young adults who experienced ACE were faced with during COVID-19. Considering what is known about our study population’s susceptibility to maladaptive coping in the face of stress, it is perhaps not surprising that participants reported that their alcohol and marijuana use increased during the pandemic when compared to their pre-pandemic consumption. *The Transactional Model of Stress and Coping*, (Lazarus & Folkman, 1987) which states that increased stress may lead to maladaptive coping mechanisms due to an individual’s perception and appraisal of threat, provides a framework for why young adults who experienced ACE may feel an intensified level of stress in traumatic conditions, such as a pandemic. The stressors of the pandemic led to using substance use as a coping mechanism (Arterberry et al., 2023; Emery et al., 2021; Horigian et al., 2021; Struik et al., 2024). In particular, some young adults indicated that anxiety and depression were reasons for wanting to use substances during the pandemic (Arterberry et al., 2023; Horigian et al., 2021; Struik et al., 2024), and some even mentioned that they were unsure of the order of use, that is if they were alleviating their mental health or using substances first. This may be due to the fact that substance use and mental health implications can overlap in young adults, especially in stressful situations.

Young adults also indicated that they used substances as a means to offset boredom (Pakdaman & Clapp, 2021), as activities were limited during the pandemic. Prolonged social distancing during the pandemic appeared to limit opportunities for social engagement and structured activities, which may have increased boredom and contributed to the use of substances as a coping strategy to fill time. Some participants also indicated that substance use was a coping mechanism for grief or losses experienced during the pandemic (Pitman et al., 2020). Corroborating our finding, Lee and Neimeyer administered the Pandemic Grief Scale and found that adults experiencing relatively high grief and loss were using more substances, likely as a coping mechanism for pandemic-related grief and loss (McManus & Ball, 2020).

Our participants also highlighted changes in healthcare delivery. Namely, many participants indicated that they utilized telehealth for mental health treatment during the pandemic (Nicholas et al., 2021; Rosenthal et al., 2021). Some young adults felt that telehealth was not the same as in-person as the experience was different for them in the way the therapy resonated with them (Ierardi et al., 2022), although others found telehealth to be relatively convenient and therefore reduce barriers to obtaining care. Future pandemic responses should focus efforts on maintaining accessible and innovative mental healthcare measures, especially for children and adolescents at risk for trauma(Fegert et al., 2020), prior to their young adult years. An interesting finding is that young adults who utilized mental healthcare reported that their substance use and mental health symptoms subsided as they found an outlet during this time (Ierardi et al., 2022). However, other participants stopped going to therapy altogether, due to several factors, such as cost, lack of insurance and hesitancy to engage in virtual platforms for therapy. Some young adults who wanted to seek therapy during this time were unable to do so because of poor finances and a lack of health insurance (Rosenthal et al., 2021). This is yet another example of how the pandemic highlighted, and perhaps even exacerbated, existing socioeconomic disadvantages (Bambra et al., 2020), and also brings to light a lack of access to care during traumatic experiences.

Conversely, some young adults described discovering new avenues for treatment or self-help during the pandemic, including group therapy and other mobile mental health applications. Rather than reflecting simple changes in service availability, this finding suggests that participants actively engaged in adaptive coping by seeking out alternative resources when traditional supports were disrupted. Consistent with *The Transactional Model of Stress and Coping* (Lazarus & Folkman, 1987), these behaviors may reflect problem-focused coping and social support-seeking strategies, as well as the use of multiple coping resources to manage pandemic-related stress. In line with this interpretation, a systematic review and meta-analysis among college students found that meditation-based interventions were effective in alleviating symptoms of anxiety and depression, underscoring the potential benefit of self-directed and technology-mediated coping strategies (González-Valero et al., 2019; Reangsing et al., 2023; Zuo et al., 2023).

Our study did have some strengths and limitations. One strength was that we had a geographically diverse sample spread across the United States. We were also able to focus our sample only on young adults who had experienced ACE, which is a population that traditionally has been at high risk for mental health problems and substance use. This study has several limitations that should be considered when interpreting the findings. First, recall bias may have influenced participants’ accounts, as young adults were asked to retrospectively describe their mental health, substance use, and mental healthcare behaviors prior to the COVID-19 pandemic. Participants’ recollections may have been shaped by current experiences or the passage of time, potentially affecting the accuracy of comparisons between pre-pandemic and pandemic periods. Second, social desirability bias represents a limitation, as participants may have underreported or modified sensitive information related to mental health symptoms, substance use, or healthcare utilization despite assurances of confidentiality. Third, selection bias is a limitation as the young adults who agreed to participate might not have been representative of all young adults with ACE. Lastly, the reliance on self-reported data limits the ability to verify mental health diagnoses, treatment engagement, or substance use behaviors, as medical records or objective measures were not available to corroborate participants’ reports. Additionally, there is a lack of self-report data on demographics such as ethnicity and race, limiting representativeness and generalizability of these results. Together, these limitations may affect the precision of the findings; however, the qualitative design remains valuable for capturing participants’ perceptions and lived experiences.

## Conclusion

The COVID-19 pandemic was a collective, global trauma. Our findings can inform future pandemic responses for young adults who have a history of ACE. We found that young adults who experienced ACE, who are already susceptible to maladaptive coping mechanisms reported increased depression, anxiety, and alcohol and marijuana use during this time. Thus, providing viable resources that are accessible, adaptable to technology and supportive of young adults with childhood trauma may be important for future directions within the field. Participation in mental healthcare services was said to help improve substance use and mental health symptomology. However, a lot of young adults also indicated that they could not access mental healthcare services due to cost and lack of insurance. Therefore, making mental healthcare more affordable and accessible can be beneficial for future preventive measures in general and for pandemic responses. Additionally, future pandemic prevention efforts should include intervening with mental healthcare during traumatic times.

## Supplementary Information

Below is the link to the electronic supplementary material.

Supplementary Material 1 (DOCX 36.0 KB)

## Data Availability

The authors will not be sharing their data due to confidentiality concerns for the study participants.
