# Supplementary material for: Substance Use, Mental Health and Healthcare Among Young Adults with Adverse Childhood Experiences During COVID-19
Source: J Child Adolesc Trauma. 2026 Jun 29;19(4):2249–60. doi: 10.1007/s40653-026-00923-1 (PMC13737248; doi:10.1007/s40653-026-00923-1)
Supplement: Supplementary file 1 [file 40653_2026_923_MOESM1_ESM.docx]

**Supplemental Table 1**

Interview Protocol Questions

| No. | Interview Question |
| --- | --- |
| — | Thank you for joining our study. I saw that you read the informational consent form and replied to the email indicating that you had read it. We previously asked whether you had any questions upon confirming that you read the informational consent form; however, before beginning this interview, do you have any additional questions regarding the study? Please say 'skip' for any questions you do not wish to answer for any reason. |
| 1 | Please state your first name. This should be a pseudonym that is different from your real name for confidentiality purposes. |
| 2 | Please state your city and state. |
| 3 | Please state your age and gender. |
| 4 | Are you currently enrolled in college? |
| 5 | Are you currently employed? If yes, please specify whether you are employed part-time or full-time. |
| 6 | Have you experienced an adverse childhood experience (ACE)? An ACE is defined as a negative event that occurs before a person reaches 18 years of age. I am now going to ask a series of questions regarding ACEs. |
| 7 | Have you experienced abuse (emotional, physical, or sexual)? |
| 8 | Have you experienced neglect (physical or emotional)? |
| 9 | Have you experienced domestic violence? |
| 10 | Have you experienced substance misuse by a member of your household? |
| 11 | Have you experienced divorce or separation of your parents or caregivers? |
| 12 | Have you experienced mental illness in a member of your household? |
| 13 | Have you experienced having a member of your household go to prison? |
| — | Mental Health During the COVID-19 Pandemic |
| 14 | Did you experience prolonged feelings of hopelessness, sadness, and/or lethargy during the COVID-19 pandemic? |
| 15 | Please elaborate on whether you experienced these feelings before the pandemic as well. |
| 16 | How often did you feel depressed between March 2020 and the present? |
| 17 | Were there certain points during the pandemic when you felt more depressed or less depressed? |
| 18 | Did you experience prolonged feelings of anxiousness, restlessness, and worry during the COVID-19 pandemic? |
| 19 | Please elaborate on whether you experienced these feelings before the pandemic as well. |
| 20 | How often did you experience anxiety between March 2020 and the present? |
| 21 | Were there certain points during the pandemic when you felt more anxious or less anxious? |
| — | Mental Healthcare During the COVID-19 Pandemic |
| 22 | During the pandemic, did you receive mental healthcare services? If no, skip to Question 26. |
| 23 | Please describe your mental healthcare experiences. |
| 24 | Did you have health insurance that covered mental healthcare during this time, or did you pay out of pocket? |
| 25 | If yes, how did you receive mental healthcare services (e.g., telehealth, in-person, or other methods)? |
| 25a | What was your experience with in-person services while wearing masks or with virtual telehealth services and limited privacy? |
| 26 | If you received mental healthcare before the pandemic, how do your current mental healthcare experiences differ from your prepandemic experiences? |
| — | Substance Use During the COVID-19 Pandemic |
| 27 | Do you use alcohol? |
| 28 | Do you use marijuana? |
| 29 | Do you use illicit drugs? |
| 30 | Do you use prescription drugs that were not prescribed to you? |
| — | Instruction: If 'yes' to 27–30, ask 31–34. If 'no', skip to 35. |
| 31 | During the pandemic, if you consumed alcohol, how often did you drink, and how many drinks did you typically consume in one sitting? Please elaborate on the context (e.g., with others or alone). |
| 31a | How did your alcohol consumption compare with your use before March 2020? |
| 32 | During the pandemic, if you used marijuana or THC products, how often did you use them, and how many milligrams did you typically consume in one sitting? Please elaborate on the context (e.g., with others or alone). |
| 32a | How did your marijuana (THC) use compare with your use before March 2020? |
| 33 | During the pandemic, if you used illicit drugs, how often did you use them, and how many milligrams did you typically consume in one sitting? Please elaborate on the context (e.g., with others or alone). |
| 33a | How did your illicit drug use compare with your use before March 2020? |
| 34 | During the pandemic, if you misused prescription drugs, how often did you do so? Please elaborate on context. |
| 34a | How did your prescription drug misuse compare with your misuse before March 2020? |
| 35 | Please explain whether you ever used substances to alleviate feelings of anxiety before and during the pandemic. |
| 36 | Please explain whether you ever used substances to alleviate feelings of depression before and during the pandemic. |
| 37 | If yes to 27–30, describe how the pandemic influenced substance use. |
| 38 | Please describe any trends in your substance use during the pandemic. |
| 38a | Please describe how these trends may be correlated with your mental health. |
| 38b | Please describe how these trends may be correlated with your mental healthcare experiences. |
| — | Stress, Resilience, and Fear During the Pandemic |
| 39 | Please describe whether you felt more stressed or more resilient during the pandemic. |
| 40 | Please explain any fears you have related to the COVID-19 pandemic. |
